# The Seasonal Variation of the Chemical Composition of Essential Oils from *Porcelia macrocarpa* R.E. Fries (Annonaceae) and Their Antimicrobial Activity

**DOI:** 10.3390/molecules181113574

**Published:** 2013-11-01

**Authors:** Erica Biolcati P. da Silva, Marisi G. Soares, Bruna Mariane, Marcelo A. Vallim, Renata C. Pascon, Patricia Sartorelli, João Henrique G. Lago

**Affiliations:** 1 Instituto de Ciências Ambientais, Químicas e Farmacêuticas, Universidade Federal de São Paulo, Diadema 09972-270, SP, Brazil; E-Mails: ericabiolcati@gmail.com (E.B.P.S.); bruna_mariane@yahoo.com.br (B.M.); marcelo.vallim@gmail.com (M.A.V.); renata.pascon@gmail.com (R.C.P.); psartorelli@unifesp.br (P.S.); 2 Instituto de Química, Universidade Federal de Alfenas, Alfenas 37130-000, MG, Brazil; E-Mail: marisigs@gmail.com

**Keywords:** *Porcelia macrocarpa*, essential oils, seasonal variation, antimicrobial activity, *C. neoformans*

## Abstract

This study investigates the impact of seasonal variation on the chemical composition of essential oils from the leaves of *Porcelia macrocarpa* (Annonaceae) obtained over the course of one year (January–December 2011) and the chemical composition of the essential oils obtained from the ripe fruits of the same plant. Furthermore, the essential oils of the leaves were investigated with respect to their antimicrobial activity. The essential oils of the leaves contain a mixture of monoterpenes, one diterpene and several sesquiterpenes. The main components were identified as the sesquiterpenes germacrene D (29%–50%) and bicyclogermacrene (24%–37%). No significant variation was observed for the composition of the essential oil of the leaves over the course ofthe year, except for the month of November, when the ripe fruit were collected. In this month, substantially decreased concentrations of germacrene D (28.8 ± 0.8%) and bicyclogermacrene (23.9 ± 0.6%) were measured and the emergence of spathulenol (10.4 ± 0.2%) was observed. The essential oils extracted from the ripe fruit revealed the presence of a variety of monoterpenes, sesquiterpenes and hydrocarbons. The main constituents of these oils were neryl (8.8 ± 0.2%) and geranyl (27.3 ± 0.7%) formates, γ-muurolene (10.3 ± 0.9%) and dendrolasin (8.23 ± 0.06%). The antimicrobial activity of the essential oil obtained from the leaves of *P. macrocarpa* towards a range of bacterial and yeast strains was examined. In order to determine the minimum inhibitory concentration (MIC) of essential oils obtained from the January collection of the leaves, broth microdilution assays were carried out, which showed a significant antimicrobial activity towards *Cryptococcus neoformans* serotypes A and D as well as *C. gattii* serotypes B and C.

## 1. Introduction

*Porcelia macrocarpa* R.E. Fries (Annonaceae) is a tree found in the forest regions on the Atlantic coast and in the interior of Brazil [1]. The chemical composition of various parts of *P. macrocarpa* has already been the subject of several scientific studies. High contents of acetylene acetogenins were found in the extracts from seeds [2], while several amides, lignanamides, and alkaloids were isolated from branch extracts [3,4,5]. The branches also contained several interesting polar compounds, such as flavonoids, trimethylamonium salts and amino acids [6]. Essential oils extracted from the leaves were analyzed by GC/MS which allowed the identification of nine individual components, of which bicyclogermacrene (27.5%) and germacrene D (37.8%) were the major components [7]. A desirable cytotoxic activity against human tumor cells was observed for these essential oils [8].

Essential oils are not only used in several therapeutic applications of folk medicine, but they also form part of a variety of modern pharmaceutical remedies [9]. However, a meticulous qualitative as well as quantitative analysis of these essential oils is of the utmost importance, since the quality of the macroscopic remedy is highly sensitive towards several factors, e.g., climatic conditions, the chemo- and biotype as well as the phenology of the plant [10]. Important biological activities have already been reported for essential oils of several species of Annonaceae [11] and our group has investigated the composition and biological activity of essential oils obtained from several other Brazilian plants [12,13,14]. The promising results of these studies encouraged us to examine the influence of the seasonal variation on the chemical composition of the essential oils of the leaves of *P. macrocarpa.* Furthermore, we were interested in a comparison between the chemical composition of these essential oils with the chemical composition of essential oils obtained from the ripe fruits of the same plant and an examination of the antimicrobial activity of leaves oil.

## 2. Results and Discussion

### 2.1. Microclimatic Factors

**Figure 1:**
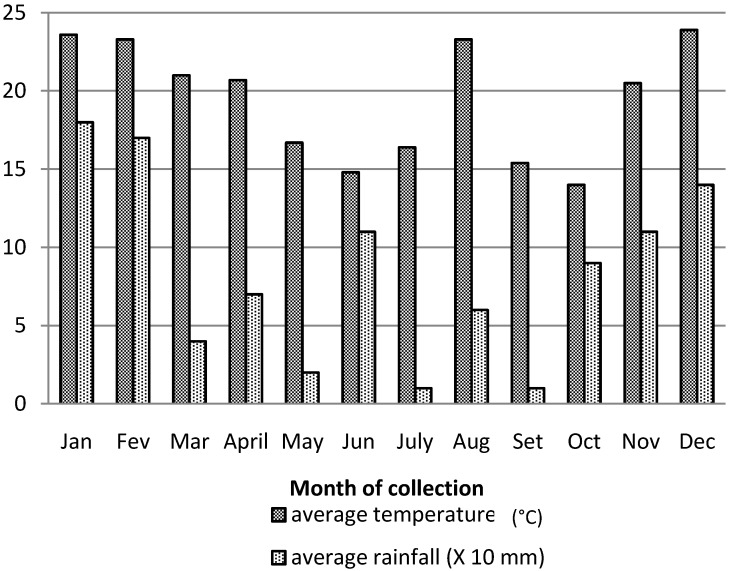
Average monthly temperatures (°C) and precipitation (×10 mm) values in 2011 (collection period).

Generally, the temperature pattern for Sao Paulo city can be divided into two periods: a colder period from ca. April–October and a warmer period from November–March. The precipitation pattern is inversely proportional to the temperature profile and minimum rainfall can usually be observed between the months of April–September. The temperature for the collection period of 2011 (see Figure 1) basically follows these temperature/precipitation averages, except for August 2011, which was unusually hot, and June 2011, which showed unusually high precipitation [15].

For the collection period of the leaves of *P. macrocarpa*, the average temperature of the warmer period (January–April and November–December) was 22.2 °C. The average temperature of the colder period from May–October was 16.7 °C. The precipitation pattern showed a maximum around December–February (monthly average of 150 mm) and a minimum around May, July and September (average of 10–20 mm).

### 2.2. Extraction Yields of the Essential Oils from the Leaves of *P. macrocarpa*

**Figure 2:**
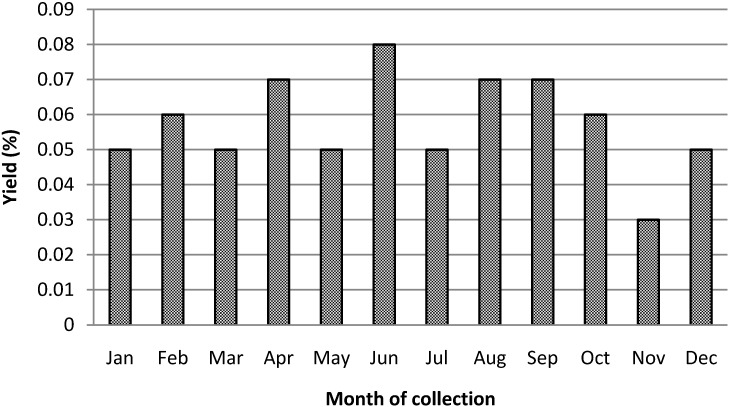
Monthly extraction yields of the essential oils from the leaves of *Porcelia macrocarpa* (January–December/2011).

Essential oils were extracted from the leaves by hydro-distillation as described in the Experimental section (Section 3.4) and the extraction yields are summarized in Figure 2. We found that extraction yields of essential oils from the leaves of *P. macrocarpa* are subject to seasonal changes, reflected in high yields obtained during the months of April (0.07 ± 0.04%), June (0.08 ± 0.02%), August (0.07 ± 0.01%) and September (0.07 ± 0%). Interestingly, the extraction yield decreased in November (0.03 ± 0%), which typically coincides with the month of fructification for this species. An impact of environmental factors on the yields of essential oils was also observed for several species such as *Rosmarinus officinalis* [16], *Mentha suaveolens* [17], *Satureja horvatii* [18], and *Baccharis trimera* [19]. The influence of several other climatic factors such as temperature and pluviometric index onto the extraction yield also needs to be taken into consideration [10]. For example, the observed precipitation during January and February was significantly higher compared to the other months of 2011. In contrast to that, the temperature remained relatively constant over the course of the study, except for the months of May, June and October. As shown in Figure 2, the maximum oil yields were obtained around June and minimum yields were obtained in December/January. This would suggest an inversely proportional relation between oil extraction yields and the temperature and precipitation pattern. Similar findings have been reported by Gazim *et al*. [20], who showed that the amount of several volatile compounds produced by *Tetradenia riparia* (Lamiaceae) were affected by temperature, humidity and rainfall changes over the course of different seasons. Moreover, Lago *et al*. reported that the relative amounts of essential oils from the leaves of *Pittosporium undulatum* could be better correlated to microclimatic parameters such as temperature and precipitation index, than to the phenology of the studied species [21]. The yield of essential oils from the fruit of *P. macrocarpa*, obtained during November 2011 was 0.05 ± 0.01% and is comparable to the average yield (0.06 ± 0.02%) obtained from the leaves (see Figure 2).

### 2.3. Chemical Composition of the Essential Oils Obtained from the Leaves and the Ripe Fruit of *P. macrocarpa*

The crude essential oils obtained from the leaves and the ripe fruits of *P. macrocarpa* were analyzed by GC (DB-5 capillary column) and GC-MS. Individual compounds were assigned according to their Kovats indices in conjunction with a comparison of the experimentally obtained mass spectra to those described in library (NIST 107) and in the literature [22]. In the essential oils of the leaves, nine individual components were observed and identified as verbanyl acetate (monoterpene), α-copaene, *iso*-longifolene, β-cedrene, α-guainene, germacrene D, bicyclogermacrene, and spathulenol (sesquiterpenes) as well as phytol (diterpene). The main compounds were germacrene D (28.8 ± 0.8%–49.6 ± 0.7%) and bicyclogermacrene (23.9 ± 0.6%–36.8 ± 0.5%). The sum of these nine components accounted for between 66.6 ± 0.1% (November) and 91.1 ± 0.6% (April) of the total oil content (see Table 1). The overall composition was similar to previously described samples [7,8]. A significant decrease (*p* < 0.05) of the relative amounts of germacrene D (28.8 ± 0.8%) and bicyclo-germacrene (23.9 ± 0.6%) was detected during November 2011, concomitant with the emergence of spathulenol (10.4 ± 0.2%), which could not be detected in any other month. As reported by Bülow and Köning, the presence of spathulenol and the reduced amounts of germacrene D and bicyclogermacrene could be partially explained by biochemical factors, such as the enzymatic oxidation of the latter compounds to form spathulenol [23]. This process could therefore be related directly to the phenology of *P. macrocarpa*, since it coincides with the end of the fructification period. However, it is worth pointing out here, that during the sterile period, the relative amounts of germacrene D and bicyclogermacrene were also non-constant (67 ± 7% in February to 84 ± 2% in April), which could be attributed to microclimatic factors, e.g., temperature and precipitation [24]. As shown in Table 1 and Figure 2, the sum of relative proportion of both sesquiterpenes was lower in January, February and December (71 ± 7%, 67 ± 7% and 78.9 ± 0.7%, respectively) compared to the other months. For these months, relatively high precipitation values (14–18 mm) and temperatures (23–24 °C) were observed. These observations suggest an influence of these microclimatic factors on the production and/or accumulation of germacrene D and bicyclogermacrene in the crude essential oils.

The essential oils from the ripe fruit consisted of 65 individual components (Table 2), which accounted for 99.6 ± 0.9% of the volatile components. The dominant compounds were monoterpenes (ca. 45%), especially neryl (8.8 ± 0.2%) and geranyl (27.3 ± 0.7%) formates. Other major fractions consisted of sesquiterpenes, such as γ-muurolene (10.3 ± 0.9%), δ-cadinene (2.44 ± 0.03%) and dendrolasin (8.23 ± 0.06%) as well as hydrocarbons such as hexacosane (6.02 ± 0.06%) and heptacosane (2.13 ± 0.06%).

### 2.4. Antimicrobial Activity

Several species of the Annonaceae are known to produce essential oils which display antimicrobial activity, e.g., *Annona vepretorum* [25], *Duguetia lanceolata* [26], *Guatteriopsis blepharophylla*, *G. friesiana*, and *G. hispida* [27]. In order to determine the antimicrobial activity of the essential oils obtained from the leaves and the ripe fruit of *P. macrocarpa*, the minimum inhibitory concentration (MIC concentration range: 0.003–1.0 mg/mL) was ascertained for the prokaryote and eukaryote microbes. No biological activity could be observed for the essential oils obtained from the fruit, since no inhibition was detected at 1.0 mg/mL. The essential oils from the leaves (collected in January 2011) however, displayed significant biological activity towards all four *Cryptococcus* strands tested. No biological activity was detected for the prokaryotes tested as well as for the several *Candida* spp. or *S. cerevisiae*. The most sensitive strand is *C. neoformans* serotype D, which exhibited a growth inhibition rate of 85% at a dose of 0.06 mg/mL essential oil (see Table 3). Another clinically very important strain is *C. neoformans* serotype A, which accounts for most of the cases of cryptococosis found among immunosuppressed patients. The essential oils of the leaves showed an inhibition rate of 80% against this strain for a dose of 0.5 mg/mL essential oil. The remaining strains of *C. gattii* serotypes B and C are pathogens, which usually affect immunocompetent patients, and required higher concentrations of around 1.0 mg/mL essential oil in order to cause inhibition rates of 98% and 61%, respectively. *C. gattii* presented a lower inhibition at the maximum concentration tested and not the traditional 80% inhibition, however, we found important to report this result. The observed MICs for all four strains were in accordance with literature values [28,29], suggesting a high accuracy of the assay. Fluconazole was used as positive control.

**Table 1:** Seasonal variation of the chemical composition of essential oils obtained from the leaves of *Porcelia macrocarpa* (monthly collection from January to December 2011).

	Relative amount (%) ^b^												
Compound ^a^	KI	January	February	March	April	May	June	July	August	September	October	November	December
verbanyl acetate	1340	0.39 ± 0.04 ^a^	0.43 ± 0.01 ^a^	0.40 ± 0.01 ^a^	0.46 ± 0.06 ^a^	0.51 ± 0.02 ^a^	0.42 ± 0.01 ^a^	0.42 ± 0.04 ^a^	0.41 ± 0.03 ^a^	1.76 ± 0.96 ^a^	0.34 ± 0.01 ^a^	0.31 ± 0.19 ^a^	0.38 ± 0.01 ^a^
α-copaene	1376	2.0 ± 0.3 ^a^	1.6 ± 0.9 ^a^	2.05 ± 0.07 ^a^	2.2 ± 0.2 ^a^	1.9 ± 0.8 ^a^	1.95 ± 0.02 ^a^	2.01 ± 0.09 ^a^	1.8 ± 0.2 ^a^	2.4 ± 0.9 ^a^	1.71 ± 0.01 ^a^	0.4 ± 0.2 ^b^	2.07 ± 0.05 ^a^
*iso*-longifolene	1387	1.4 ± 0.1 ^a^	1.5 ± 0.1 ^a^	1.9 ± 0.1 ^a^	2.0 ± 0.9 ^a^	1.8 ± 0.9 ^a^	0.9 ± 0.8 ^b^	0.50 ± 0.02 ^b^	0.53 ± 0.02 ^b^	1.8 ± 0.9 ^a^	1.2 ± 0.7 ^b^	0.6 ± 0.2 ^b^	0.23 ± 0.01 ^b^
β-cedrene	1418	0.72 ± 0.06 ^a^	0.80 ± 0.07 ^a^	0.75 ± 0.01 ^a^	0.9 ± 0.1 ^a^	1.3 ± 0.4 ^b^	1.4 ± 0.4 ^b^	1.08 ± 0.02 ^b^	1.1 ± 0.1 ^b^	0.96 ± 0.01 ^a^	0.85 ± 0.09 ^a^	0.6 ± 0.3 ^a^	0.85 ± 0.02 ^a^
α-guaiene	1439	0.9 ± 0.3 ^a^	1.1 ± 0.3 ^a^	0.71 ± 0.01 ^a^	0.56 ± 0.03 ^b^	0.52 ± 0.07 ^b^	0.61 ± 0.08 ^b^	0.43 ± 0.01 ^b^	0.61 ± 0.02 ^b^	0.61 ± 0.01 ^b^	0.4 ± 0.2 ^b^	0.41 ± 0.07 ^b^	0.89 ± 0.01 ^a^
germacrene D	1480	40 ± 7 ^a^	39 ± 7 ^a^	46.3 ± 0.4 ^a^	47 ± 1 ^a^	49.6 ± 0.7 ^b^	47.1 ± 0.9 ^a^	49 ± 2 ^b^	45 ± 1 ^a^	46.6 ± 0.1 ^a^	43 ± 3 ^a^	28.8 ± 0.8 ^b^	46.2 ± 0.7 ^a^
bicyclogermacrene	1494	31 ± 3 ^a^	28 ± 7 ^a^	34.2 ± 0.3 ^a^	37 ± 1 ^a^	34 ± 2 ^a^	36.8 ± 0.5 ^a^	32 ± 2 ^a^	30.8 ± 0.2 ^a^	32.5 ± 0.1 ^a^	35 ± 3 ^a^	23.9 ± 0.6 ^b^	32.7 ± 0.3 ^a^
spathulenol	1576	0	0	0	0	0	0	0	0	0	0	10.4 ± 0.2	0
phytol	1955	7.3 ± 0.9 ^a^	18 ± 2 ^b^	3.2 ± 0.2 ^c^	1.2 ± 0.3 ^d^	0.55 ± 0.05 ^d^	0.45 ± 0.04 ^d^	0.6 ± 0.2 ^d^	0	0.7 ± 0.5 ^d^	0.6 ± 0.2 ^d^	1.24 ± 0.04 ^d^	0.55 ± 0.01 ^d^
monoterpenes		0.39 ± 0.04	0.43 ± 0.01	0.40 ± 0.01	0.46 ± 0.06	0.51 ± 0.02	0.42 ± 0.01	0.42 ± 0.04	0.41 ± 0.03	1.76 ± 0.96	0.34 ± 0.01	0.31 ± 0.19	0.38 ± 0.01
sesquiterpenes		76 ± 3	72 ± 3	85.9 ± 0.2	89.4 ± 0.7	88.6 ± 0.6	88.7 ± 0.4	85 ± 1	79.5 ± 0.6	84.8 ± 0.5	82 ± 1	65.0 ± 0.3	80.7 ± 0.5
diterpene		7.3 ± 0.9	18 ± 2	3.2 ± 0.2	1.2 ± 0.3	0.55 ± 0.05	0.45 ± 0.04	0.6 ± 0.2	0	0.7 ± 0.5	0.6 ± 0.2	1.24 ± 0.04	0.55 ± 0.01
**TOTAL**		**84 ± 2**	**90 ± 3**	**89.6 ± 0.1**	**91.1 ± 0.6**	**90 ± 1**	**89.6 ± 0.4**	**89 ± 1**	**79.9 ± 0.4**	**86.8 ± 0.3**	**83.7 ± 0.7**	**66.6 ± 0.1**	**82.1 ± 0.3**

^a^ Individual compounds were assigned according to their Kovats indices in conjunction with a comparison of the experimentally obtained mass spectra to those described in library (NIST 107) and in the literature [22]; ^b^ Values in the same line with different subscript (a, b, c, and d) are significantly different within months of collection; t: (*p* < 0.05). All values displayed represent the mean value ± standard deviation of three independent experiments. Statistical analyses were performed by analysis of variance (ANOVA) using the BIOESTAT 5.0 (Stat Soft Inc., Tulsa, OK, USA) software package. A probability value of *p* < 0.05 was considered statistically significant.

**Table 2:** The chemical composition of essential oils obtained from the ripe fruit of *Porcelia macrocarpa* (November 2011).

Compound ^a^	KI	*Relative amount (%)* ^b^
*o*-cymene	1026	0.09 ± 0.01
benzene acetaldehyde	1042	0.15 ± 0.02
γ-terpinene	1059	0.18 ± 0.06
oct-2*E*-en-1-ol	1066	0.12 ± 0.02
dehydrolinalool	1090	0.06 ± 0.01
non-3*Z*-en-1-ol	1157	0.06 ± 0.01
terpinen-4-ol	1177	0.09 ± 0.02
methyl salicylate	1191	0.12 ± 0.03
dec-2*E*-enal	1263	0.09 ± 0.02
geranial	1267	0.18 ± 0.01
neryl formate	1282	8.8 ± 0.2
geranyl formate	1298	27.3 ± 0.7
dimethoxy-*Z*-citral	1318	0.13 ± 0.05
dimethoxy-*E*-citral	1341	1.26 ± 0.03
ethyl nerolate	1354	0.99 ± 0.01
*Z*-α-damascone	1358	0.66 ± 0.02
neryl acetate	1361	0.84 ± 0.01
α-ylangene	1375	1.14 ± 0.01
geranyl acetate	1381	0.51 ± 0.02
β-bourbonene	1388	1.12 ± 0.02
*E*-α-damascone	1393	0.75 ± 0.03
ethyl geranate	1395	0.21 ± 0.01
*E*-caryophyllene	1419	1.14 ± 0.01
β-duprezianene	1422	0.56 ± 0.06
neryl acetone	1436	0.50 ± 0.03
*E*-β-farnesene	1456	2.8 ± 0.7
γ-muurolene	1479	10.3 ± 0.9
α-amorphene	1484	0.63 ± 0.03
*cis*-eudesma-6,11-diene	1489	1.17 ± 0.03
α-muurolene	1500	0.69 ± 0.01
butylated hydroxytoluene	1515	0.96 ± 0.01
δ-cadinene	1523	2.44 ± 0.03
*trans*-cadina-1,4-diene	1534	0.3 ± 0.02
α-cadinene	1538	0.40 ± 0.02
α-calacorene	1545	0.27 ± 0.01
*E*-nerolidol	1563	1.03 ± 0.05
dendrolasin	1571	8.23 ± 0.06
globulol	1590	1.08 ± 0.03
viridiflorol	1592	0.33 ± 0.01
cubeban-11-ol	1595	0.21 ± 0.01
geranyl 2-methylbutanoate	1601	0.48 ± 0.01
geranyl isovalerate	1607	0.30 ± 0.03
5-*epi*-7-*epi*-α-eudesmol	1607	0.48 ± 0.02
himachalol	1653	0.78 ± 0.01
α-cadinol	1654	0.92 ± 0.01
*E*-bisabol-11-ol	1667	0.45 ± 0.02
γ-dodelactone	1677	0.8 ± 0.1
*Z*-nerolidyl acetate	1677	0.45 ± 0.01
α-bisabolol	1685	0.99 ± 0.07
davanol acetate	1689	0.30 ± 0.01
2*E*,6*E*-farnesol	1743	0.15 ± 0.02
β-bisabolenal	1769	0.15 ± 0.01
1-octadecene	1790	1.6 ± 0.7
*n*-hexadecanol	1875	1.38 ± 0.01
5*E*,9*E*-farnesyl acetone	1913	0.18 ± 0.03
isophytol	1947	0.30 ± 0.03
3*Z*-cembrene A	1966	0.63 ± 0.01
ethyl hexadecanoate	1993	0.54 ± 0.02
*E,E*-geranyl linalool	2027	0.21 ± 0.06
manool	2057	0.48 ± 0.04
*n*-octadecanol	2077	0.39 ± 0.02
*E*-phytol acetate	2218	1.14 ± 0.02
pentacosane	2500	1.86 ± 0.03
hexacosane	2600	6.02 ± 0.06
heptacosane	2700	2.13 ± 0.06
monoterpenes		44.8 ± 0.9
sesquiterpenes		37.1 ± 0.9
diterpenes		0.51 ± 0.06
hydrocarbons		10.49 ± 0.06
other compounds		6.7 ± 0.1
TOTAL		99.6 ± 0.9

^a^ Individual compounds were assigned according to their Kovats indices in conjunction with a comparison of the experimentally obtained mass spectra to those described in library (NIST 107) and in the literature [22]; ^b^ Values displayed represent the mean value ± standard deviation of three independent experiments.

**Table 3:** Minimum inhibitory concentrations (MICs) obtained from broth microdilution assays for essential oils from the leaves of *Porcelia macrocarpa* (January 2011).

Microorganism	Essential oil dosage (mg/mL)	Positive Control
		Fluconazole (mg/mL)
*C. neoformans* (serotype A)	0.50 (80 ± 18%)	0.013
*C. neoformans* (serotype D)	0.06 (95 ± 8%)	0.006
*C. gattii* (serotype B)	1.00 (98 ± 6%)	0.025
*C. gattii* (serotype C)	1.00 (61 ± 1%) *	0.006

Numbers in parenthesis represent the mean percentage inhibition at each MIC ± standard deviation. * 61% inhibition does not represent MIC_80_ in this case.

In addition to the desirable antimicrobial activity, previous reports have also reported a beneficial antifungal activity for some monoterpenes, diterpenes and sesquiterpenes (especially germacrene D and bicyclogermacrene), which were found in the essential oils of other plants [30,31,32,33]. For example, Cabral *et al*. showed that germacrene D, the main compound in *Vitex rivularis* oil, displayed a significant activity against dermatophytes [34]. Bicyclogermacrene and α-copaene have moreover been associated with an antifungal activity, mainly in studies involving the genus *Candida* and dermatophytes [31,34,35,36,37]. To the best of our knowledge, there are no reports in the scientific literature describing the activity of the essential oils (or their main components) of the leaves of *P. macrocarpa* against *C. neoformans* and *C. gattii*, which cause fungal meningitisin immunocompromised as well as immunocompetent patients [38,39].

## 3. Experimental

### 3.1. Chemical Reagents

All solvents used were of analytical grade and purchased from CAAL (São Paulo, Brazil). Linear *n*-alkane (C_8_–C_20_) reference standards, as well as all culture media and standard antibiotic discs of fluconazole and chloramphenicol were obtained from Sigma-Aldrich Chemical Co. (St. Louis, MO, USA). All other chemicals were purchased from Merck (Darmstadt, Germany), except for hygromycin B, which was obtained from Invitrogen (Carlsbad, CA, USA).

### 3.2. Microclimatic Factors

Temperatures were measured *in situ* with a digital Pocket Weather Meter Kestrel 3000 (Nielsen-Kellerman, Boothwyn, PA, USA). Precipitation values (in mm) were measured during each period of collection (from 12th to 18th day) using a pluviometer, which was custom-made in our laboratory.

### 3.3. Plant Material

Leaves of *P. macrocarpa* R.E. Fries (Annonaceae) were collected randomly from three individual trees in the Jardim Botânico de São Paulo (São Paulo, SP, Brazil) at 12 a.m. on the 15th day of each month (January to December 2011). Ripe fruit samples were collected from the very same trees on 15th November, 2011 at 12 a.m. Reference specimen were deposited at the herbarium of the Instituto de Botânica (São Paulo, Brazil) and compared with those under reference SP76791. Samples of the crude oils are available from the authors.

### 3.4. Hydro-Distillation of the Essential Oils

Each batch of fresh leaves or fruitsamples from *P. macrocarpa* was hydro-distilled for four hours in a Clevenger type apparatus [21]. The essential oils were extracted from the aqueous fraction using CH_2_Cl_2_ (3 × 5 mL). The combined organic fractions were subsequently dried over anhydrous Na_2_SO_4_, before the solvent was evaporated and the oil was stored at 4 °C in the absence of light. 

### 3.5. Gas Chromatography Analysis (GC)

The crude essential oils were analyzed by GC, using a Shimadzu GC-2010 gas chromatograph, equipped with an FID-detector and an automatic injector (Shimadzu AOC-20i). As the stationary phase, an RtX-5 capillary column (5% phenyl, 95% polydimethylsiloxane, 30 m × 0.32 mm × 0.25 μm film thickness; Restek, Bellefonte, PA, USA) was used with helium as the carrier gas (flow rate: 1 mL/min). The oven temperature was raised from 60 °C to 280 °C at a rate of 3 °C/min and subsequently kept at 280 °C for further ten minutes. The injector temperature was 220 °C and the detector (FID) was kept at 280 °C. Composition percentages were obtained from electronic integration of the FID output and a series of linear *n*-alkanes (C_8_–C_20_), which were used as reference points for the determination of the Kovats indices (KI).

### 3.6. Gas Chromatography- Mass Spectrometry (GC-MS) Analysis

GC-MS analysis was carried out using a Shimadzu GC-17A chromatograph connected to a MS-QP-5050A mass spectrometer. The GC analysis was carried out with an RtX-5 capillary column (5% phenyl, 95% polydimethylsiloxane, 30 m × 0.32 mm × 0.25 μm film thickness; Restek, Bellefonte, PA, USA) and the operating conditions were identical with those described in the previous section. Retention indices for all compounds were determined according to the Kovats indices (KI), as described in the previous section. The EI-MS analysis was carried out under an ionization voltage of 70 eV and an ion source temperature of 230 °C. The identification of individual compounds was achieved by a comparison of the KI values in conjunction with matching mass spectrometric fragmentation patterns to those of mass spectra library (NIST 107), published MS fragmentation patterns [22] and/or MS spectra of authentic compounds.

### 3.7. Microbial Strain Media, Antibiotics and Growth Conditions

In order to test the antimicrobial activity of the essential oils obtained from the leaves and fruits of *P. macrocarpa*, Gram-positive (*Streptococcus equi*—CBMAI 264, *Staphylococcus epidermidis*—CBMAI 604, and *Enterococcus fecalis*), Gram-negative (*Escherichia coli*, *Serratia marcescens*—CBMAI 469, and *Pseudomonas aeruginosa*—CBMAI 602) and yeast (*Candida dubliniensis*—ATCC 7978, *C. tropicalis*—ATCC 13803, *C. albicans*—ATCC 18804/CBMAI 560, *C. glabrata*—ATCC 90030, *C. parapsilosis*—clinical isolate 68, *C. krusei*—clinical isolate 9602, *Cryptococcus neoformans*—KN99α serotype A/JEC21 serotype D, *C. gattii*—NIH312 serotype C/R265 serotype B, and *Saccharomyces cerevisiae*—BY4742) strains were subjectedto a broth microdilution assays. Microbial strains were kept as criostocks at −80 °C, cultivated onYEPD (2% peptone, 2% dextrose and 2% agar) platesfor yeasts (1% yeast extract) or LB (1% tryptone, 1% NaCl and 2% agar) for bacterial strains (0.5% bacterial strains). Fluconazole and chloramphenicol were used as positive controls for yeast and bacteria, respectively. Essential oils were diluted to 10% with dimethylsulfoxide (DMSO).

### 3.8. Broth Microdilution Assay for the Determination of Minimum Inhibitory Concentrations (MIC)

Micro titer plates (96 wells) were used for broth microdilution assays in order to ascertain the MIC for each tested strain. Two independent assays were conducted according to the guidelines of the *National Committee for Clinical Laboratory Standards* (CLSI, M100-S9). The following minor modifications were implemented: the target microorganisms were grown in test tubes overnight in 3 mL of the respective medium (RPMI 1640 for yeast and BHI for bacteria) at 30 °C and agitated in a rotary shaker (150 rpm). The cellular concentration was adjusted to 1× 10^2^ – 2 × 10^2^ CFU (yeast) and 1 × 10^4^ – 2 × 10^4^ CFU (bacteria) per well. The concentration was confirmed by viability counts on YEPD and LB plates. Nine dilutions of essential oils and reference standards were used (two-fold serial dilutions). A negative sterilization control, containing medium only and a positive growth control, containing cells and 10 μL DMSO (100% growth) were also included. Microtiter plates were subsequently incubated at 30 °C for 24 or 48 h, respectively. Finally, the absorbance at 530 nm was measured in a plate reader (Epoch, Bio-Tek, Winooski, VT, USA). The threshold for the MIC was set at a minimum of an 80% growth inhibition. All tests were performed in triplicate in 100 µL of reaction volume. The concentration range for the essential oils of the leaves ranged from 0.003–1.0 mg/mL. Fluconazole and chloramphenicol concentrations ranged between 0.0007 and 0.05 mg/mL and 0.00312 and 0.400 mg/mL, respectively.

## 4. Conclusions

A great benefit of this study is the characterization of the composition of the essential oils of the leaves and ripe fruits of *P. macrocarpa*. The biological activity documented in this study should be of great pharmacological interest, since the major components of the essential oils may now be tested individually against both species of the *Cryptococcus* genus, which continue to cause life-threatening diseases and demand new potent antifungal drugs in order to allow effective treatment.
